# Specialized naphthoquinones present in *Impatiens glandulifera* nectaries inhibit the growth of fungal nectar microbes

**DOI:** 10.1002/pld3.132

**Published:** 2019-05-13

**Authors:** Anna K. Block, Elena Yakubova, Joshua R. Widhalm

**Affiliations:** ^1^ Center for Medical, Agricultural and Veterinary Entomology U.S. Department of Agriculture‐Agricultural Research Service Gainesville Florida; ^2^ Department of Horticulture and Landscape Architecture Purdue University West Lafayette Indiana; ^3^ Center for Plant Biology Purdue University West Lafayette Indiana

**Keywords:** allelopathy, *Impatiens glandulifera* (Himalaya balsam), naphthoquinone, nectar

## Abstract

The invasion success of *Impatiens glandulifera* (Himalayan balsam) in certain parts of Europe and North America has been partially attributed to its ability to compete for bee pollinators with its rich nectar and due to its capacity to produce and release allelopathic 1,4‐naphthoquinones (1,4‐NQs) from its roots and leaves. Given that other 1,4‐NQs present in the digestive fluids of certain carnivorous plants are proposed to control microbial colonization, we investigated the potential for the 1,4‐NQs, 2‐methoxy‐1,4‐naphthoquinone (2‐MNQ) and lawsone, to fulfill an analogous role in the nectaries of *I. glandulifera*. Both 2‐MNQ and lawsone were detected in the floral nectaries of *I. glandulifera* at levels comparable to leaves and roots, but were discovered to be at significantly higher levels in its extra‐floral nectaries (EFNs) and to be present in EFN nectar itself. Nectar microbe inhibition assays revealed that the common nectar bacteria *Gluconobacter oxydans* and *Asaia prunellae* are not inhibited by 2‐MNQ or lawsone, although both compounds were found to inhibit the growth of the common fungal nectar microbes *Metschnikowia reukaufii* and *Aureobasidium pullulans*. Taken together, these findings suggest that 2‐MNQ and lawsone could serve to protect the rich nectar of *I. glandulifera* against fungal growth. The high abundance of 2‐MNQ and lawsone in *I. glandulifera* EFNs may also point to an unsuspected mechanism for how allelopathic 1,4‐NQs are leached into the soil where they exhibit their known allelopathic effects.

**Abbreviations**
1,4‐NQ: 1,4‐naphthoquinone
2‐MNQ: 2‐methoxy‐1,4‐naphthoquinone
EFNs: extra‐floral nectaries

## 1 INTRODUCTION

*Impatiens glandulifera* Royle is known for its thin pin‐shaped extra‐floral nectaries (EFNs) covering its shoot nodes, leaf petioles, and basal leaf teeth (Lüttge, 2013). Native to the western Himalaya, *I. glandulifera* is a tall annual plant that was introduced as an ornamental in Europe and North America in the 19th century (Beerling & Perrins, 1993). It has since become naturalized along waterways and in forests, and is considered extremely invasive in certain areas due to its impact on native organisms through several means, including changing soil characteristics and microclimate (Ruckli, Rusterholz, & Baur, 2013), disrupting arbuscular mycorrhizal symbiosis (Ruckli, Rusterholz, & Baur, 2014), and competing for bee pollinators with its strongly scented and nectar‐rich flowers (Chittka & Schürkens, 2001).

**Figure 1:**
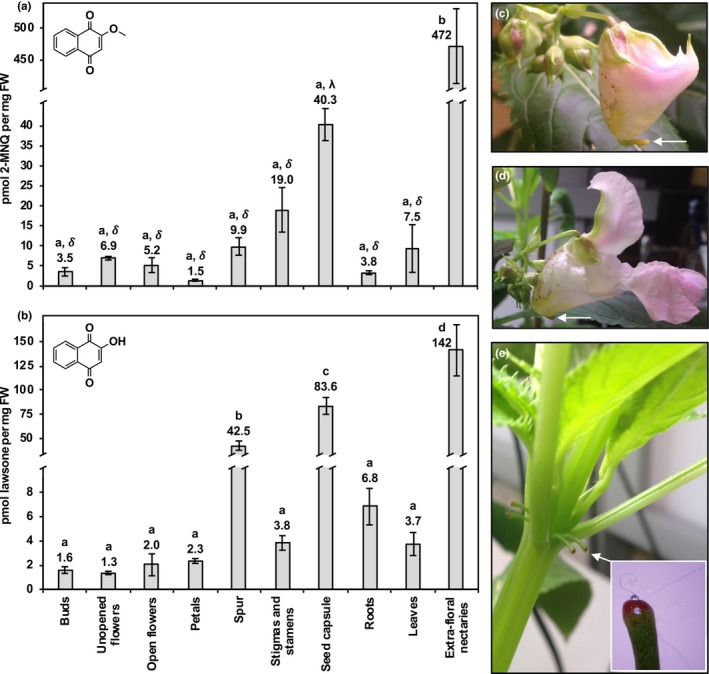
Targeted profiling of specialized naphthoquinones in *Impatiens glandulifera* organs. Pool sizes of 2‐methoxy‐1,4‐naphthoquinone (2‐MNQ) (a) and lawsone (b). Note that open flowers included tissues from petals, spurs, reproductive parts, receptacles, and sepals. Images of unopened (c) and open (d) *I. glandulifera* flowers with white arrowhead pointing to floral nectaries (spurs). Image of *I. glandulifera* shoot with white arrow and inset showing an example of a sampled extra‐floral nectary (EFN) (e). All data are means ± *SEM* (*n* = ≥3 biological replicates). Different English alphabet letters above bars indicate significant differences via analysis of variance (ANOVA) followed by a post hoc Tukey HSD test (α = 0.05). Due to the greater than 10‐fold difference in 2‐MNQ pool sizes between EFNs versus all other organs, a second statistical test was performed to examine differences in mean 2‐MNQ pool sizes between all organs excluding EFNs. For this planned comparison, the more conservative Bonferroni method was used. Different Greek letters above bars indicate significant differences via ANOVA followed by a post hoc Bonferroni test (α = 0.001) between all samples except EFNs

Recent evidence indicates that releasing the inhibitory molecule 2‐methoxy‐1,4‐naphthoquinone (2‐MNQ, Figure 1a) from its leaves and roots has played a major role in the invasion success of *I. glandulifera* (Ruckli, Hesse, Glauser, Rusterholz, & Baur, 2014). The physiological mechanism by which 2‐MNQ is deployed remains unknown, but it may involve leaching from the plant surface by rain (Ruckli, Hesse, et al., 2014). 2‐MNQ belongs to a diverse class of natural products called the specialized 1,4‐naphthoquinones (1,4‐NQs) which include the allelochemicals juglone and shikonin (Widhalm & Rhodes, 2016). Like juglone (Rudnicka, Polak, & Karcz, 2014) and shikonin (Durán et al., 2017), 2‐MNQ inhibits germination and growth of herbaceous plants (Ruckli, Hesse, et al., 2014). Mycelium inhibition assays revealed that 2‐MNQ additionally elicits negative effects on the growth of ectomycorrhiza fungi (Ruckli, Hesse, et al., 2014). The presence of 2‐MNQ in leaves may also confer protection against insect feeding by inhibiting the conversion of the molting hormone ecdysone to its more physiologically active form 20‐hydroxyecdysone (Mitchell, Brescia, Smith, & Morgan, 2007). In addition to 2‐MNQ, another 1,4‐NQ, lawsone, is present in the roots and flowering aerial parts of *I. glandulifera* and other *Impatiens* species (Lobstein et al., 2001). Although it has not been reported to elicit inhibitory effects on fungi and other plants like 2‐MNQ, lawsone is cytotoxic against certain bacteria (Sauriasari et al., 2007).

Specialized 1,4‐NQs, including plumbagin and droserone, produced by the carnivorous plants *Dionaea muscipula* (Venus flytrap) and *Nepenthes* sp. (pitcher plants) are found in high abundance in the digestive fluids secreted by these organisms (Chan, Hong, Yin, & Chan, 2016; Culham & Gornall, 1994). These 1,4‐NQs are thought to play a role in maintaining sterility of the digestive fluids to reduce competition for prey‐derived nutrients or in promoting a specific microbiome that contributes activities for digesting captured insects (Buch et al., 2013; Ogihara et al., 2013). Outside of carnivorous plants, it remains unclear if specialized 1,4‐NQs play similar roles in influencing microbial growth in other plant fluids, such as nectar. Therefore, given that *I. glandulifera* produces specialized 1,4‐NQs and contains nectar‐rich flowers and EFNs, we investigated if 2‐MNQ and lawsone are present in *I. glandulifera* nectaries and if these 1,4‐NQs are capable of inhibiting the growth of common nectar microbes. The results from this study provide evidence supporting an additional role for 2‐MNQ and lawsone, and illuminate a potential unpredicted mechanism for deploying these allelochemicals into the rhizosphere.

## 2 METHODS

### 2.1 Plant growth conditions and general experimental procedures

Seeds of *I. glandulifera* were collected near Durham, England and cold stratified for 1 month at 4°C on rockwool cubes moistened with tap water. Germinated seedlings were transferred to AeroGarden 7 LED Indoor Garden units (Scotts Miracle‐Gro Company, Marysville, OH, USA) and grown hydroponically with acidified water supplemented with 50% strength water‐soluble fertilizer (20N‐1.3P‐15.8K; ICL Specialty Fertilizers, Dublin, OH) to provide the following (in mg/L): 75 N, 4.9 P, 59.5 K, 6 Mg, 10.5 S, 0.75 Fe, 0.2 Mn and Zn, 0.1 Cu and B, and 0.05 Mo. Nitrate and ammoniacal sources of nitrogen were provided as 61% and 39% total N, respectively. Irrigation water was supplemented with 93% sulfuric acid (Brenntag, Reading, PA) at 0.08 mg/L to reduce alkalinity to 50 mg/L and pH to a range of 5.8–6.2. Nutrient solutions were changed weekly. Unless otherwise mentioned, all reagents were from Fisher Scientific (Pittsburgh, PA, USA).

### 2.2 Naphthoquinone profiling

Extraction and analysis of 1,4‐NQs were performed in dimmed light to reduce potential photodegradation. Up to 300 mg of flash‐frozen *I. glandulifera* organs (flower buds, unopened flowers, open flowers, petals, stamens and stigmas, spurs, seed capsules, leaves, and EFNs with visible nectar) from 3‐month‐old flowering plants were extracted in 5 ml 100% v/v methanol for 16 hr with moderate shaking. Approximately 1 g of harvested roots from hydroponically grown plants were allowed to dry in between two pieces of filter paper in the dark at room temperature for 10 min prior to flash freezing and methanol extraction. Quantification of 2‐MNQ and lawsone was achieved by filtering 1 ml of the methanolic extract through a 0.2‐μm PTFE syringe filter and directly analyzing up to 50 μl on an Agilent 1260 Infinity II high‐performance liquid chromatography (HPLC) system (Palo Alto, CA, USA) coupled with diode array detection. Injected samples were separated using a Zorbax SB‐C18 column (5‐μm, 4.6 × 250 mm; Agilent) connected to a Zorbax SB‐C18 analytical guard column (5‐μm, 4.6 × 12.5 mm; Agilent). Mobile phases were (A) 10 mM ammonium formate in 0.1% formic acid (v/v) in HPLC‐grade water and (B) 0.1% formic acid (v/v) in acetonitrile. After sample injection, the column was eluted with 5% B for 2 min, followed by a linear gradient to 95% B over 15 min, then an isocratic hold at 95% B for 8 min, a 3 min ramp back to 5% B, and a 7 min isocratic hold of 5% B at a 0.3 ml/min flow rate and 40°C column temperature. Lawsone and 2‐MNQ were detected spectrophotometrically at 244 nm. Retention times were 22.5 and 24.5 min for lawsone and 2‐MNQ, respectively. Compounds were quantified according to external calibration curves generated from different amounts of authentic standards. Lawsone and 2‐MNQ were both purchased from Sigma‐Aldrich (St. Louis, MO, USA).

### 2.3 Nectar microbe inhibition assays

Due to the lack of information regarding the microbial composition of *I. glandulifera* nectaries, nectar microbe inhibition assays carried out in this study were performed using the common nectar fungi *Metschnikowia reukaufii* and *Aureobasidium pullulans* (GenBank IDs: MF319536 and MF325803) as described in Rering, Beck, Hall, McCartney, & Vannette (2017), and the common nectar bacteria *Gluconobacter oxydans* and *Asaia prunellae*. Plates were made containing 20 ml of sterile synthetic nectar (0.3% w/v sucrose; 0.1% w/v peptone; 0.6% w/v each of glucose and fructose; 0.1 mM each of glycine, l‐alanine, l‐asparagine, l‐aspartic acid, l‐glutamic acid, l‐proline, and l‐serine, and 2% w/v agar). Actively growing cultures (5 ml) at 1 × 10^7^ cells/ml in 0.3% w/v sterile agar were layered on the surface of the plates and allowed to dry. Plates were marked to divide the area into half, and a sterile 50‐mm^2^ filter paper disk was placed at the center of each half and 10 μl of the desired concentration of 2‐MNQ or lawsone in DMSO was pipetted onto the filter paper disk and allowed to dry. Plates were placed at 30°C overnight and the inhibition zone measured.

## 3 RESULTS AND DISCUSSION

Many species within the genus *Impatiens* are known to produce 1,4‐NQs (Hook, Mills, & Sheridan, 2014), and recent evidence suggests that these compounds function to chemically mediate interactions with other plants and fungi in the rhizosphere (Ruckli, Hesse, et al., 2014). The observation by Lobstein et al. (2001) that lawsone and 2‐MNQ are present in *I. glandulifera* flowers therefore led us to hypothesize that these 1,4‐NQs serve an additional role by influencing the growth of nectar microbes. To test this hypothesis, we first profiled the spatial abundance of lawsone and 2‐MNQ in various *I. glandulifera* organs using HPLC coupled with diode array detection. Like previous reports (Lobstein et al., 2001; Ruckli, Hesse, et al., 2014), both 2‐MNQ and lawsone were observed in roots, leaves, and whole flowers (Figure 1a,b). The pool sizes of 2‐MNQ and lawsone in flower buds, unopened flowers, and open flowers were found to range from 1.3 to 6.9 pmol/mg FW (Figure 1a,b). Further dissection of open flowers revealed that both 2‐MNQ and lawsone are present in similar amounts in petals and the stamens and stigmas (Figure 1a,b). Previous examinations of *Nepenthes* sp. found that the 1,4‐NQ plumbagin is present in the wax layer of pitcher traps (Raj, Kurup, Hussain, & Baby, 2011) where it is thought to provide antifungal activity against phytopathogenic fungi spread by visiting insect prey (Shin, Lee, & Cha, 2007). The large flowers of *I. glandulifera* completely enclose their bee pollinators, forcing the backs of the insects to contact the stigmas and stamens located on the roof of the flower (Beerling & Perrins, 1993). Therefore, it can be envisioned that the presence of 2‐MNQ and lawsone in *I. glandulifera* flower petals, stamens, and stigmas may have a similar effect as plumbagin in *Nepenthes* sp. pitcher traps to reduce the spread of pathogenic fungi potentially spread by pollinators.

**Figure 2:**
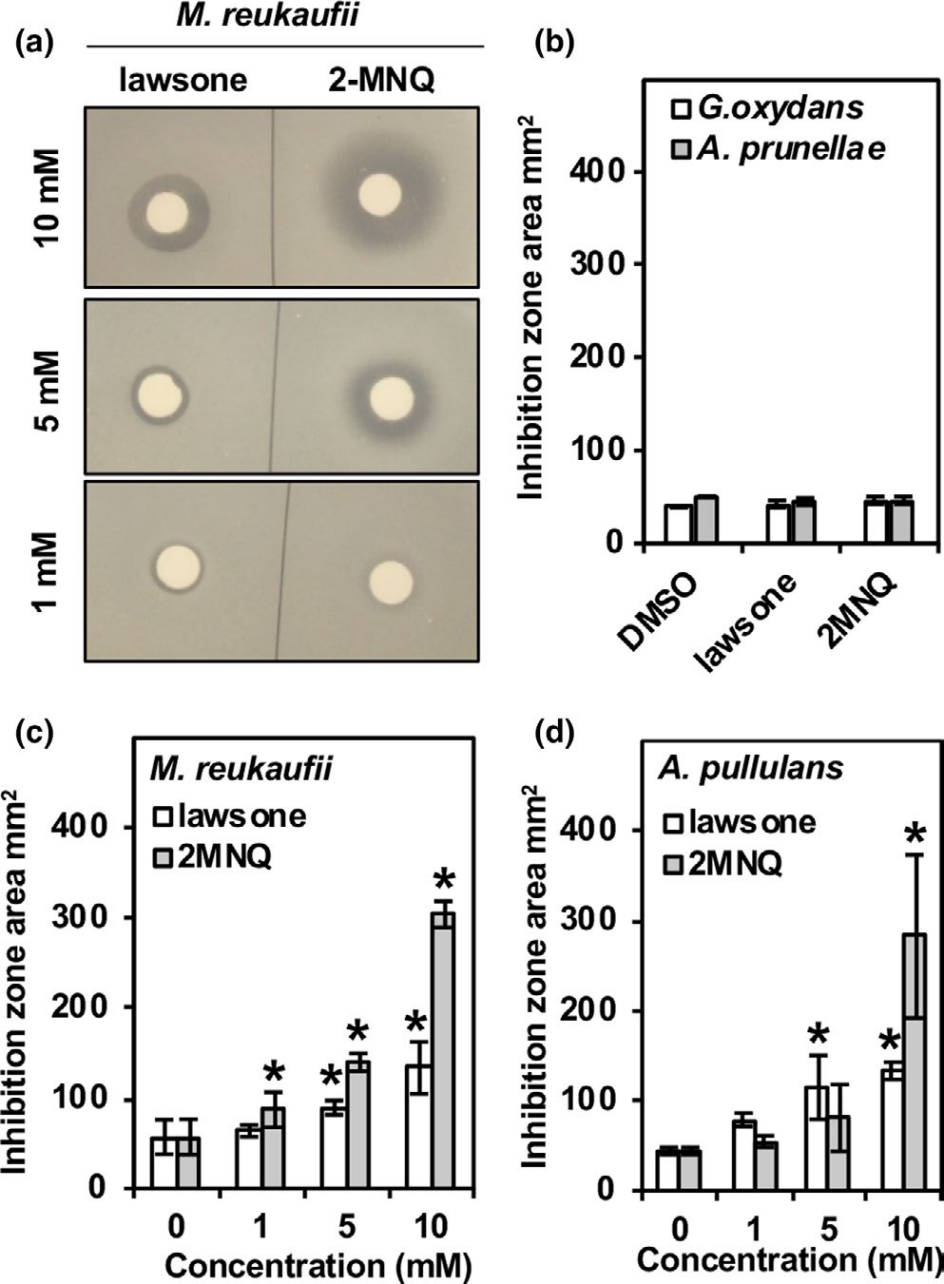
Antimicrobial activity of lawsone and 2‐MNQ against nectar microbes. Representative images of inhibition zone clearing in an antimicrobial assay using lawsone and 2‐MNQ on the nectar microbe *Metschnikowia reukaufii* (a). Inhibition zone areas of *Gluconobacter oxydans* and *Asaia prunellae* treated with 10 mM of lawsone, 2‐MNQ, or a DMSO control (b). Inhibition zone areas of *M. reukaufii* treated with various concentrations of lawsone and 2‐MNQ (c). Inhibition zone areas of *Aureobasidium pullulans* treated with various concentrations of lawsone and 2‐MNQ (d). (*) indicates treatments significantly different from DMSO control (*p* ≤ 0.05 by a pair wise *t* test, *n* = 3)

The nectar located in *I. glandulifera* flowers is secreted into the end of the calyx‐spur located at the back of the flower (Figure 1c,d) (Beerling & Perrins, 1993). Measurement of 1,4‐NQs in the spur of open flowers revealed that while 2‐MNQ is present at levels similar to roots, leaves, and other flower parts, lawsone is present at a significantly higher level, 42.5 pmol/mg FW (Figure 1a,b). Although lawsone has not yet been reported to elicit the inhibitory effects on fungi observed with 2‐MNQ, it was demonstrated to be cytotoxic against certain bacteria (Sauriasari et al., 2007). To determine if the production of lawsone and 2‐MNQ can impact the growth of nectar microbes, the ability of these compounds to inhibit the growth of two bacterial and two fungal nectar microbes was examined. Using growth inhibition assays on artificial nectar agar plates the inhibition zone produced by each compound was measured (Figure 2). Neither of the bacterial species tested, *G. oxydans* or *A. prunellae,* were inhibited by 10 mM of lawsone or 2‐MNQ (Figure 2b). For the two fungal microbes, *M. reukaffi* and *A. pullulans*, both lawsone and 2‐MNQ inhibited growth versus the control when applied at 10 mM (Figure 2c–d). At a concentration of 5 mM, only lawsone affected *A. pullulans* growth relative to the control (Figure 2d). Conversely, at a concentration of 1 mM, growth of *M. reukaffi* was not sensitive to lawsone but was inhibited by 2‐MNQ relative to control (Figure 2c). Taken together, these data suggest that at sufficient levels lawsone and 2‐MNQ both elicit antifungal activity.

Altering the levels of secondary metabolites in nectar can have an impact on the nectar microbiome. For instance, transgenic *Nicotiana attenuata* plants with silenced putrescine‐*N*‐methyl transferase, that do not produce nicotine and have different pyridine‐alkaloid composition, have drastically different bacterial composition and diversity in their floral nectar when compared to their isogenic parents (Aizenberg‐Gershtein et al., 2015). In addition, in vitro antimicrobial growth assays have shown that the plant derived secondary metabolites found in nectar such as caffeine and nicotine have general antimicrobial activity, whereas catapol displays species specific responses (Vannette & Fukami, 2016). Plants may actively regulate their nectar microbiome not only to suppress potentially pathogenic microbes but also to promote the growth of beneficial ones. Nectar microbiomes can influence a variety of nectar properties including temperature, pH, and total sugar concentrations (Herrera, García, & Pérez, 2008; Herrera & Pozo, 2010; Vannette, Gauthier, & Fukami, 2012). It was recently shown that nectar microbes such as the fungi *M. reukaffi* produce volatile compounds that can affect honey bee preference, thereby potentially impacting plant–pollinator interactions (Rering et al., 2017). Given the antifungal activities observed here with 2‐MNQ and lawsone (Figure 2), it is therefore possible that if accumulated in high enough concentrations in nectar the presence of these 1,4‐NQs in spurs of *I. glandulifera* flowers could affect the growth and composition of the nectar microbiome. Whether lower concentrations than those tested here have a biologically meaningful impact on influencing nectar microbial growth remains to be seen.

**Figure 3:**
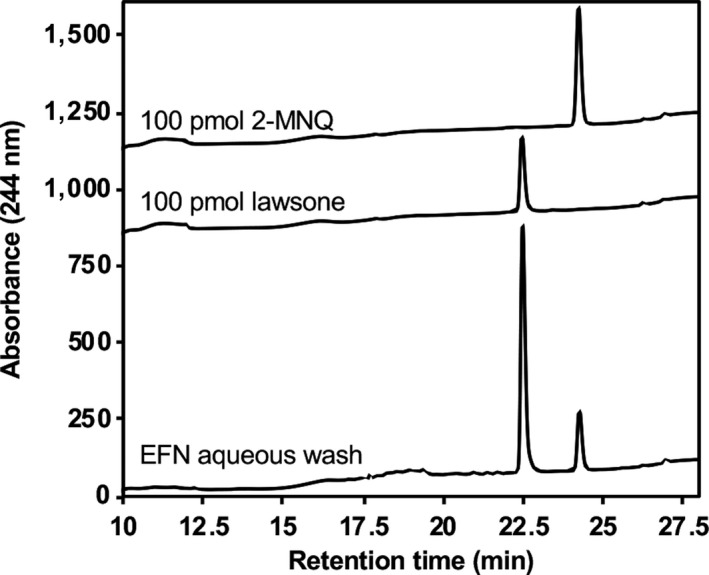
Detection of lawsone and 2‐methoxy‐1,4‐naphthoquinone (2‐MNQ) in nectar from *Impatiens glandulifera* extra‐floral nectaries (EFNs). Five EFNs collected from *I. glandulifera* were dipped in 1 ml HPLC‐grade water to wash off visible nectar droplets (see Figure 1e inset). The resulting wash was concentrated to 100 μl and 25 μl was directly analyzed by HPLC with diode array detection to look for the presence of lawsone and 2‐MNQ. Traces corresponding to 100 pmol of authentic lawsone and 2‐MNQ standards have been offset for clarity. Retention times for lawsone and 2‐MNQ were 22.5 and 24.5 min, respectively

In addition to the nectar contained in flower spurs, *I. glandulifera* produces nectar in EFNs located on its shoot nodes, leaf petioles, and basal leaf teeth (Lüttge, 2013). Nectar excreted by EFNs is hypothesized to attract beneficial insects or other animal mutalists that provide protection against herbivorous insects. As 1,4‐NQs were detected in *I. glandulifera* flower spurs, we also profiled EFNs with visible nectar isolated from shoot nodes (Figure 1e) for 2‐MNQ and lawsone. The levels of 2‐MNQ and lawsone detected in EFNs were higher than those in any other organ examined (Figure 1a,b). EFNs contained more than threefold higher levels of lawsone than those observed in spurs (142 vs. 42.5 pmol/mg FW; Figure 1b), and 47‐fold higher levels of 2‐MNQ (472 vs. 9.9 pmol/mg FW; Figure 1a). Next, to determine if the detected 1,4‐NQs are present in the nectar, we analyzed an aqueous wash collected from five *I. glandulifera* EFNs with visible nectar droplets for the occurrence of 2‐MNQ and lawsone. Indeed, both compounds were highly abundant in the analyzed nectar wash (Figure 3). Interestingly, lawsone appeared to be more prevalent than 2‐MNQ. This could suggest that lawsone is more highly secreted than 2‐MNQ into nectar, though it may also reflect a higher aqueous solubility. If one assumes that (a) the nectar volume of an EFN is similar to that in an *I. glandulifera* floral nectary (780 nl) (Barrow & Pickard, 1984), (b) an EFN collected from a shoot node (Figure 1e) weighs approximately 1 mg, and (c) that all of the 1,4‐NQs measured in EFNs (Figure 1a,b) are present in nectar, then it can be estimated that the concentration of lawsone and 2‐MNQ in EFN nectar approaches 0.2 and 0.6 mM, respectively. Although these values are lower than the concentrations of lawsone and 2‐MNQ found in this study to exhibit antifungal activity (Figure 2c,d), it is important to note that the reported concentrations from the inhibition assays correspond to those in the 10 μl solutions applied to filter paper disks (Figure 2a). The effective concentration eliciting antifungal activity is considerably lower given the compound diffuses from the point of origin outwardly into the media. Thus, it seems reasonable to predict that lawsone and 2‐MNQ may be present at high enough concentrations, especially considering the sum or synergy of their effects, to influence the microbiome of EFN nectar. Interestingly, a recent study by Ruckli, Hesse, et al. (2014) concluded that the invasion success of *I. glandulifera* may be due in part to the inhibitory effects of 2‐MNQ in the rhizosphere after the compound is leached from its leaves into the soil by rainwater. How 2‐MNQ is exuded from leaves to be leached still remains an open question; however, given the relative high abundance of 1,4‐NQs in EFNs (Figure 1a,b), and their presence in the nectar itself (Figure 3), it should be further investigated if EFNs serve as the conduit by which 2‐MNQ and lawsone are released from aerial tissues into the surrounding environment.

Of the organs examined, the second highest production of 1,4‐NQs after the EFNs was found in fresh seed pod capsules, with 2‐MNQ and lawsone at 40.3 and 83.6 pmol/mg FW, respectively (Figure 1a,b). Given the inhibitory effect of 2‐MNQ on ectomycorrhiza fungi (Ruckli, Hesse, et al., 2014), and that of 2‐MNQ and lawsone on nectar fungi (Figure 2), the high concentrations of 1,4‐NQs in the seed capsule could act to provide antifungal protection to developing seeds.

## 4 CONCLUSION

Naphthoquinones produced by *I. glandulifera* play multiple roles in plant–biotic interactions. Here, we provide evidence that 2‐MNQ and lawsone are contained in the floral and EFNs of *I. glandulifera* and that these compounds exhibit antifungal activity that may contribute to influencing the nectar microbiome. Moreover, the high abundance of 2‐MNQ and lawsone in EFNs may point to an unsuspected role for this organ in releasing 1,4‐NQs into the environment.

## CONFLICT OF INTEREST

The authors declare no conflict of interest with this study.

## AUTHOR CONTRIBUTIONS

AKB and JRW conceived the project, analyzed the data, and wrote the paper. AKB, EY, and JRW performed experiments. All authors read, edited, and approved the final manuscript.
