# Supplementary material for: Specialized naphthoquinones present in Impatiens glandulifera nectaries inhibit the growth of fungal nectar microbes
Source: Plant Direct. 2019 May 13;3(5):e00132. doi: 10.1002/pld3.132 (PMC6589542; doi:10.1002/pld3.132)
Supplement: Supplementary file 1 [file PLD3-3-e00132-s001.docx]

**Author responses to reviewer comments about Block *et al.* “Specialized naphthoquinones present in Impatiens glandulifera nectaries inhibit the growth of fungal nectar microbes”**

Reviewer #1: 
In this study, Block et al. found the amount of two specialized metabolites - 2-methoxy-1,4- naphthoquinones (2-MNQ) and lawsone, both belonging to the 1,4-naphthoquinone (1,4-NQs) family - are the highest in extra-floral nectaries, seeds and capsule and nectar spurs in the many Impatiens glandulifera organs they have tested. These compounds show anti-fungal activities against Metschnikowia reukaufii and Aureobasidium pullulans, but no anti-bacterial effects against Gluconobacter oxydans and Asaia prunellae. I think that this manuscript is interesting and clearly described with well-written discussions. However, there are some points to be clarified: 

1. While authors claimed they see no anti-bacterial effects against Gluconobacter oxydans and Asaia prunellae, no supporting data or figure(s) were presented in this manuscript. Nor can I find experimental information in the method section.

**Author response: Thank you for catching this. Yes, we did refer to these observations in the Results but we did not include the experimental information in the Methods section. We have fixed this by indicating that the same method was used for the fungal and bacterial assays in Methods section 2.3 as follows (please see new lines 154-158 of Tracked Changes version/lines 148-152 of Clean version):**

**“Due to the lack of information regarding the microbial composition of *I. glandulifera* nectaries, nectar microbe inhibition assays carried out in this study were performed using the common nectar fungi *Metschnikowia reukaufii* and *Aureobasidium pullulans* (GenBank IDs: MF319536 and MF325803) as described in Rering *et al*. (Rering et al., 2017), and the common nectar bacteria *Gluconobacter oxydans* and *Asaia prunellae.*”**

**We also decided to include data showing the lack of effect of lawsone and 2MNQ on the bacteria, which have been added in Figure 2 as new panel 2b. The legend for Figure 2 has also been updated accordingly (please see new lines 460-462 of Tracked Changes version/lines 393-395 of Clean version).**

2. There is consistent typo of A. pullans while I believe the authors meant to say Aureobasidium pullulans (A. pullulans). This happens throughout the manuscript, in Figure 2 and its figure legend.

**Author response: We apologize for this error and thank the reviewer for pointing it out. The typos have been corrected throughout the manuscript.**

3. Fig 2a-c: Please provide data of DMSO control.

**Author response: Data for the DMSO controls (reported as “0 mM” in new panels 2b,c,d) was added in Figure 2 panels where appropriate (please see revised Figure 2 and legend, lines 460-463 of Tracked Changes version/lines 393-396 of Clean version).**

4. Line 195-202 and Figure 2 legend: In Figure 2, authors performed statistical analysis comparing effects of compounds at certain concentration to DMSO control. However, in the main text, authors compared the efficiency of 2-MNQ and lawsone against each other. This requires a different statistical analysis than the one performed in Figure 2. Please update.

**Author response: The reviewer is correct that comparing the efficacy of 2-MNQ to lawsone requires a different statistical test than that performed in Figure 2. The purpose of these experiments was to test if lawsone and 2-MNQ can affect the growth of common nectar microbes. It was not intended to compare the effects of one compound to another, though it is true that we did do this in our discussion. Taking the point made here by Reviewer 1 into account with the last point made by Reviewer 2, we have softened our interpretation to focus on the ability of each compound to independently exhibit antimicrobial growth without comparing the efficacy of one compound to the other. With these changes to the text, the initial statistical tests performed are now appropriate for the discussion. The following text has been included as new lines 201-211 of Tracked Changes version (lines 190-200 of Clean version). We thank the reviewer for this comment.**

**“To determine if the production of lawsone and 2-MNQ can impact the growth of nectar microbes, the ability of these compounds to inhibit the growth of two bacterial and two fungal nectar microbes was examined. Using growth inhibition assays on artificial nectar agar plates the inhibition zone produced by each compound was measured (Figure 2). Neither of the bacterial species tested, *G. oxydans* or *A. prunellae,* were inhibited by 10 mM of lawsone or 2-MNQ (Figure 2b). For the two fungal microbes, *M. reukaffi* and *A. pullulans*, both lawsone and 2-MNQ inhibited growth versus the control when applied at 10 mM (Fig 2c-d). At a concentration of 5 mM, only lawsone affected *A. pullulans* growth relative to the control (Figure 2d). Conversely, at a concentration of 1 mM, growth of *M. reukaffi* was not sensitive to lawsone but was inhibited by 2-MNQ relative to control (Figure 2c). Taken together, these data suggest that at sufficient levels lawsone and 2-MNQ both elicit antifungal activity.”**

5. Fig 1a, 1b: Please consider adding y axis breaks that allows readers to focus on the smaller bars too. I would encourage designing the y axis break in a way that makes 1a and 1b easier to visually compare. It is also quite interesting that 2-MNQ in seed and capsules are statistically not different from 2-MNQ in roots given how small the error bars are. I would also suggest adding brackets to indicate the tissues that were dissected from open flowers in the x axis. Fig 1b legend is missing.

**Author response: We agree that the initial presentation of Figure 1a,b made it difficult to visually make comparisons between organs with smaller pool sizes of lawsone and/or 2-MNQ. Per the reviewer’s suggestion we have added y-axis breaks to both Figure 1a and 1b; however, different breaks were made to each panel in order to optimize visual comparisons within panels (*i.e.* comparisons between organs). To facilitate comparisons between panels (*i.e.* different compounds present in the same organ), we have provided the numerical average pool size value above each bar. We think that these changes have improved the figure, thank you. Please see revised Figure 1.**

**The reviewer also keenly pointed out that the 2-MNQ pool sizes of seed capsules compared to roots, and perhaps other organs, appear to be different even though the differences were not statistically significant by the post-hoc Tukey HSD test. To help address this, we consulted with a specialist at the Purdue Statistical Consulting Service (**[**http://www.stat.purdue.edu/scs/**](http://www.stat.purdue.edu/scs/)**; acknowledged on new lines 319-320 of Tracked Changes version/lines 266-267 of Clean version). It appears that despite an inclusive α-value of 0.05, the very large difference in mean 2-MNQ pool sizes between extra-floral nectaries (EFNs) and the rest of the analyzed organs masked the comparisons between the organs with smaller pool sizes. To address this, we were advised to perform a second statistical post-hoc test excluding EFNs from the comparison. Because this was a “planned comparison” we used the more conservative Bonferroni method, with a calculated α-value of 0.001, to compare the mean 2-MNQ pool size differences between all analyzed organs excluding EFNs. When applying this test, indeed the pool size of 2-MNQ in seed capsules is significantly larger than those in the other analyzed organs. We have included the results of the Bonferroni test in revised Figure 1a. The Figure 1 legend has been updated accordingly to reflect the second test. The Figure 1 legend now contains information about the open flower tissues sampled and the missing Figure 1b legend. Please see new lines 444-456 of Tracked Changes version (lines 377-389 of Clean version).**

6. It would be very informative if the author could provide an estimation of the biological range of 2-MNQ and lawsone in I. glandulifera nectar to give readers an idea how the anti-microbial range tested in Figure 2 is compared to biological concentration.

**Author response: This is a very interesting question and we can indeed make an estimation that suggests the biological concentrations approach the concentrations needed to elicit antifungal activity. A new Figure 3 and changes to the text have been made. Reviewers 2 and 3 alluded to something similar, so we have addressed all three reviewers’ questions as follows.**

**We first determined if lawsone and 2-MNQ are present in the nectar secreted from EFNs (more on floral nectaries below). To test this, we performed a simple aqueous wash of EFNs to strip off the visible nectar and then qualitatively profiled the wash for the presence of lawsone and 2-MNQs. Both 1,4-NQs were highly abundant in the wash fraction (HPLC traces with standards are shown as a new Figure 3) and so it was therefore concluded that lawsone and 2-MNQ are indeed present in *I. glandulifera* EFN nectars. Knowing that these compounds are in the nectar, we then made a “back-of-the-envelope” calculation using the pool sizes from Figure 1a,b with estimations of EFN nectar volumes and weights and the assumption that all detected compounds are in the nectar. We came up with a concentration of 0.2 and 0.6 mM for lawsone and 2-MNQ, respectively. While it is true that these estimates are 2-25 times lower than the concentrations reported to elicit effects in our study, they are within the range of biological significance considering the limitation of the inhibition assay (detailed in modified manuscript text shown below). Please note that in our response to Reviewer 3 that we describe our unsuccessful attempt to get a more precise determination of effective concentration to elicit growth inhibition. In addition to a new Figure 3 (please see new lines 467-473 of Tracked Changes version/lines 400-406 of Clean version for legend), we have modified the text as follows (please see new lines 274-298 of Tracked Changes version/lines 228-245 of Clean version):**

**“Next, to determine if the detected 1,4-NQs are present in the nectar, we analyzed an aqueous wash collected from five *I. glandulifera* EFNs with visible nectar droplets for the presence of 2-MNQ and lawsone. Indeed, both compounds were highly abundant in the analyzed nectar wash (Figure 3). Interestingly, lawsone appeared to be more prevalent than 2-MNQ. This could suggest that lawsone is more highly secreted than 2-MNQ into nectar, though it may also reflect a higher aqueous solubility. If one assumes that (i) the nectar volume of an EFN is similar to that in an *I. glandulifera* floral nectary (780 nL) (Barrow and Pickard, 1984), (ii) an EFN collected from a shoot node (Figure 1e) weighs approximately 1 mg and (iii) that all of the 1,4-NQs measured in EFNs (Figure 1a,b) are present in nectar, then it can be estimated that the concentration of lawsone and 2-MNQ in EFN nectar approaches 0.2 and 0.6 mM, respectively. Although these values are lower than the concentrations of lawsone and 2-MNQ found in this study to exhibit antifungal activity (Figure 2c,d), it is important to note that the reported concentrations from the inhibition assays correspond to those in the 10 µL solutions applied to filter paper discs (Figure 2a). The effective concentration eliciting antifungal activity is considerably lower given the compound diffuses from the point of origin outwardly into the media. Thus, it seems reasonable to predict that lawsone and 2-MNQ may be present at high enough concentrations, especially considering the sum or synergy of their effects, to influence the microbiome of EFN nectar.”**

**As the concentrations of lawsone and 2-MNQ appear to be lower in floral nectaries than EFNs, we have addressed the biological significance with the following modification to the text as follows (please see new lines 260-265 of Tracked Changes version/lines 214-219 Clean version):**

**“Given the antifungal activities observed here with 2-MNQ and lawsone (Figure 2), it is therefore possible that if accumulated in high enough concentrations in nectar the presence of these 1,4-NQs in spurs of *I. glandulifera* flowers could affect the growth and composition of the nectar microbiome. Whether lower concentrations than those tested here have a biologically meaningful impact on influencing nectar microbial growth remains to be seen.”**

**Thank you for bringing up these points. Addressing them has greatly enhance our manuscript.**

7. I encourage the author to add information, if there are any, regarding whether the four common microbes chosen in this work have been shown to be isolated from I. glandulifera.

**Author response: To the best of our knowledge there is no information on the microbial composition of *I. glandulifera* nectaries. We have mentioned this in the text by providing a rationale for our choice of tested microbes in Methods section 2.3: “Nectar Microbe Inhibition Assays” as follows (please see new lines 154-158 of Tracked Changes version/lines 148-152 of Clean version):**

**“Due to the lack of information regarding the microbial composition of *I. glandulifera* nectaries, nectar microbe inhibition assays carried out in this study were performed using the common nectar fungi *Metschnikowia reukaufii* and *Aureobasidium pullulans* (GenBank IDs: MF319536 and MF325803) as described in Rering *et al.* (Rering et al., 2017), and the common nectar bacteria *Gluconobacter oxydans* and *Asaia prunellae.*”**

8. Line 96: Venus flytrap

**Author response: Thank you, this has been fixed.**

9. Line 113: transferred

**Author response: Thank you, this has been fixed.**

10. Line 146-156: provide info on anti-bacterial assays

**Author response: Thank you again for catching this. This has been addressed now (see #1 above).**

Reviewer #2: 
This is a well-executed study reporting anti-microbial effects of specialized naphthoquinones. The data is clear and the presentation flows well. I just have a couple of minor comments. 

I assume the 1,4-NQ and 2MNQ are both commercially available (didn't check). It would be useful to note the source of the authentic standards and solvent used.

**Author response: Both lawsone and 2-MNQ were purchased from Sigma-Aldrich. We have indicated this in Methods section 2.2: “Naphthoquinone Profiling” as follows (please see new lines 150-151 of Tracked Changes version/lines 144-145 of Clean version):**

**“Lawsone and 2-MNQ were both purchased from Sigma-Aldrich (St. Louis, MO, USA).”**

**All other reagents came from Fisher Scientific, which is noted on new lines 123-124 of Tracked Changes version (lines 121-122 of Clean version). Thank you.**

Can the authors clarify how EFNs were collected, e.g., with or without visible nectars (as shown in Fig. 2E inset), just the tip or the entire 'pin'? Just wondering if those NQs are also present in nectars (or perhaps not soluble). Note, the panel label 'e' is missing from Figure 1 legend.

**Author response: This is an excellent question. Thank you for bringing it up. Yes, we did collect entire ENFs with their visible nectars. We have indicated this in Methods section 2.2: “Naphthoquinone Profiling” as follows (please see new lines 128-131 of Tracked Changes version/lines 126-129 of Clean version):**

**“Up to 300 mg of flash-frozen *I. glandulifera* organs (flower buds, unopened flowers, open flowers, petals, stamens and stigmas, spurs, seed capsules, leaves and EFNs with visible nectar) from three-month-old flowering plants were extracted in 5 mL 100% v/v methanol for 16 h with moderate shaking.”**

**To further answer your question, yes, it does appear as if lawsone and 2-MNQ are present in nectar. We performed a simple aqueous wash of EFNs to strip off the visible nectar and then qualitatively profiled the wash for the presence of lawsone and 2-MNQ. Both 1,4-NQs were highly abundant in the wash fraction (HPLC traces with standards are shown as a new Figure 3) and so it was therefore concluded that lawsone and 2-MNQ are indeed present in *I. glandulifera* EFN nectars. Please see the Figure 3 legend on new lines 467-473 of Tracked Changes version (lines 400-406 of Clean version). Thank you for bringing this up. We feel that addressing this has significantly improved the manuscript. Also note that in response to Reviewer 1, point #6, we have expanded on this observation to address the question of biological relevance of the compounds in EFNs and nectar.**

**The legend for Figure 1e (new line 449 of Tracked Changes version/line 382 of Clean version) has been fixed. Thank you for catching this.**

L197-202, the data for A. pullans seemed noisier, especially at 5 mM. While the description is supported by statistics, the trend does not seem to suggest that Lawsone is more potent than 2MNQ (probably no significant difference between Lawson and 2MNQ). It'd be better to soften the interpretation, noting that the responses were most evident at higher (10 mM) concentrations, again with 2MNQ showing a stronger inhibitory effect than Lawsone.

**Author response: Thank you for pointing this out. Reviewer 1, point #4, alluded to something similar. We have attempted to address both reviewers’ comments by modifying the text as follows (please see new lines 201-211 of Tracked Changes version/lines 190-200 of Clean version). We thank the reviewer for this comment.**

**“To determine if the production of lawsone and 2-MNQ can impact the growth of nectar microbes, the ability of these compounds to inhibit the growth of two bacterial and two fungal nectar microbes was examined. Using growth inhibition assays on artificial nectar agar plates the inhibition zone produced by each compound was measured (Figure 2). Neither of the bacterial species tested, *G. oxydans* or *A. prunellae,* were inhibited by 10 mM of lawsone or 2-MNQ (Figure 2b). For the two fungal microbes, *M. reukaffi* and *A. pullulans*, both lawsone and 2-MNQ inhibited growth versus the control when applied at 10 mM (Fig 2c-d). At a concentration of 5 mM, only lawsone affected *A. pullulans* growth relative to the control (Figure 2d). Conversely, at a concentration of 1 mM, growth of *M. reukaffi* was not sensitive to lawsone but was inhibited by 2-MNQ relative to control (Figure 2c). Taken together, these data suggest that at sufficient levels lawsone and 2-MNQ both elicit antifungal activity.”**

Reviewer #3: 
The authors carried out profile of the levels of naphthoquinones 2-MNQ and lawsone in different tissues of Imaptiens glandulifera. They complemented the profile study with in vitro inhibitory assays that suggest that these naphthoquinones possess antifungal activity. 

If the authors could comment on the concentrations used in the inhibition studies. The concentrations detected in the tissues were in the picomole range. How well does this correlate to the 1-10 mM concentrations used in the inhibition studies. Are the tested inhibitory concentrations biologically relevant?

**Author response: This is a very interesting question and one that Reviewers 1 and 2 alluded to as well. Please see our detailed answer in our response to Reviewer 1, point #6. Briefly, we have now provided data as a new Figure 3 showing that lawsone and 2-MNQ are present in EFN nectars. We also have made an estimation (assumptions detailed in response to Reviewer 1, point #6) that suggests the approximated biological concentrations approach the effective inhibitory concentrations found in this study. We have modified the Discussion in the text (lines 274-298 of Tracked Changes version/lines 228-245 of Clean version) to reflect this critical point. Thank you for bringing this up. We feel that addressing this point has greatly strengthened the manuscript.**

In addition, is it possible to determine an in vitro minimum inhibitory concentration of the compounds against the fungi to complement the inhibition zone study in Fig 2

**Author response: We agree that this would have been a nice complement to the current study. To assess concentrations lower than those of the limit of the inhibition zone study used here, we initially attempted growth inhibition assays in liquid culture for the fungal microbes. This would have allowed us to determine the *in vitro* minimum inhibitory concentration. Unfortunately, both microbes have a strong tendency to form biofilms in liquid culture which make them unsuited for these assays.**

**No Editor comments to address**
